# Temporal Trends in Lung Cancer Mortality Across 48 Countries from 1950 to 2019: An Ecological Study

**DOI:** 10.1177/10732748261493115

**Published:** 2026-10-07

**Authors:** Matthew T. Warkentin, Yibing Ruan, Alain Tremblay, Darren R. Brenner

**Affiliations:** 1 Department of Oncology, Cumming School of Medicine, University of Calgary, Calgary, AB, Canada; 2 Department of Cancer Epidemiology and Prevention Research, Cancer Care Alberta, Calgary, AB, Canada; 3 Department of Medicine, Cumming School of Medicine, University of Calgary, Calgary, AB, Canada

**Keywords:** epidemiology, global burden, lung cancer, mortality, population, tobacco control, trends

## Abstract

**Background:**

The widespread success of tobacco control policy and smoking cessation campaigns has led to considerable reductions in lung cancer mortality. However, it is currently not known how many more deaths might have occurred if not for the substantial progress made in lung cancer control.

**Methods:**

In this retrospective study, we used age-specific population counts and lung cancer mortality rates from 1950 to 2019 (from 48 countries) from the Global Cancer Observatory. We estimated age-standardized mortality rates (ASMR), standardized to the WHO (2000-2025) Standard Population. Peak mortality was defined as the year when the maximum ASMR was reached for each country and sex. For each year in the period following peak mortality, the expected number of lung cancer deaths was estimated using peak-year age-specific mortality rates and age-specific population counts for that year. The number of avoided deaths was estimated as the difference between expected and observed lung cancer deaths in that period.

**Results:**

Lung cancer mortality peaked for males in all 48 countries but has yet to peak for females in 11 countries. There have been 3.08 million avoided lung cancer deaths since mortality rates peaked for many countries (2.93 million for males, 0.15 million for females). Across these countries, 21.4% and 4.3% of expected lung cancer mortality has been avoided since mortality peaked for males and females, respectively. Fourteen countries have reduced lung cancer mortality more than 25% for men, while only two countries have for women. There have been statistically significant reductions in lung cancer deaths for 46 out of 48 (96%) countries for men and 14 out of 48 (29%) countries for women.

**Conclusion:**

There has been a substantial number of lung cancer deaths avoided since mortality rates peaked for many countries, though the reduction in mortality has not been observed in all countries or equally for males and females.

## Introduction

Lung cancer is the most common cancer and leading cause of cancer mortality globally,^1,2^ with an estimated nearly 2.5 million incident cases (12.4% of cancer incidence) and 1.8 million deaths (18.7% of cancer mortality) in 2022.^2-4^ Lung cancer ranks first and second in mortality among men and women, respectively and is the most common cause of cancer death in 89 out of 185 countries based on data from the Global Cancer Observatory (GLOBOCAN).^2^ Most of the geotemporal differences in lung cancer incidence rates are reflected in the patterns in tobacco-product use between countries.^3^ Historically, smoking rates were higher among countries with high human development indices (HDI).^2,3^ As of 2022, among high or very high HDI countries, the age-standardized mortality rates (per 100,000) for lung cancer were 30.1 and 11.8 in males and females, respectively, compared to 9.6 and 3.9 for low or medium HDI countries.^2^ These differences in lung cancer mortality rates are largely attributable to differences in smoking rates in these countries, though there are other notable risk factors including air pollution and other country-specific factors. In addition, there is an estimated 20-to-25-year lag between smoking rates and lung cancer rates.^2,5^

However, the widespread success of tobacco control policy (bans on advertising, promotion, marketing) and smoking cessation campaigns have led to drastic reductions in tobacco-product consumption and lung cancer rates that peaked decades ago in many countries.^6^ Due to the lag period between changes in smoking and disease onset,^5^ it can be difficult to demonstrate an impact on cancer control and prevention. It is not currently known how many more lung cancer deaths would have occurred if not for public health interventions and the considerable progress made in lung cancer control. The goal of this study was to estimate the amount of progress made in reducing lung cancer mortality and quantify the amount of avoided lung cancers deaths across 48 countries.

## Methods

### Data Sources

We performed an ecological time-series study using country-level cancer mortality and population estimates from the World Health Organization’s Global Cancer Observatory (GLOBOCAN).^2,7^ We obtained the age-specific mortality rates and age-specific population counts separately for each country and sex from 1950 to 2019 (when available). The age-specific lung cancer mortality rates and population counts were reported in five-year age groups except for the 85 or older group. Lung cancer mortality was defined based on the International Classification of Diseases (ICD, 10^th^ revision, version 2010) using codes C33 to C34.^8^ Not all data were available for both males and females or for the entire period. This study involved the analysis of publicly available data so approval by a human research ethics committee was not required.

### Statistical Analysis

We estimated the observed number of lung cancer deaths as the product of the age-specific mortality rates and the corresponding age-specific population counts. To smooth the annual variation in lung cancer mortality rates, we computed five-year rolling averages for both the age-specific and age-standardized mortality rates (ASMR). We estimated ASMR using the observed (non-smoothed) age-specific mortality rates and the World Health Organization’s World Standard Population (WHO 2000-2025).^9^ ASMR are reported per 100,000. The peak year of lung cancer mortality was selected based on the highest ASMR based on the five-year rolling average. For both the age-specific and age-standardized mortality rates, the rolling average used 5 years of data, where available, but fewer data points were used at the boundaries of the period of available data. The age-specific lung cancer mortality rates (based on the five-year rolling average) from the peak year of mortality were used to estimate the expected cancer deaths. To estimate the expected number of lung cancer deaths, we applied the peak-year age-specific mortality rates to the age-specific population counts for each year in the period following peak mortality. The number of avoided lung cancer deaths was estimated as the difference between the expected and observed number of deaths. The total number of observed, expected, and avoided lung cancer deaths are based on summations over the post-peak mortality period. Standardized mortality ratios (SMR) were estimated as the ratio of the observed and expected lung cancer deaths (observed/expected). Confidence intervals (95%) for the SMR were estimated using the Vandenbroucke method.^10^ The annual relative reduction in lung cancer deaths in the period following peak mortality was estimated as the ratio of the avoided and expected lung cancer deaths. Lung cancer mortality data were not available for all countries and for all years. Missing data were excluded from all analyses. For some countries, peak lung cancer mortality was at the earliest year of the observed data. The true peak may have been earlier and thus our estimates of avoided deaths may be conservative for those countries. A schematic describing the data collection and statistical approach used in this study is presented in Figure 1. All statistical analyses were performed using R version 4.2.1 (2022-06-23). The reporting of this study conforms to the STROBE guidelines.^11^

**Figure 1.**
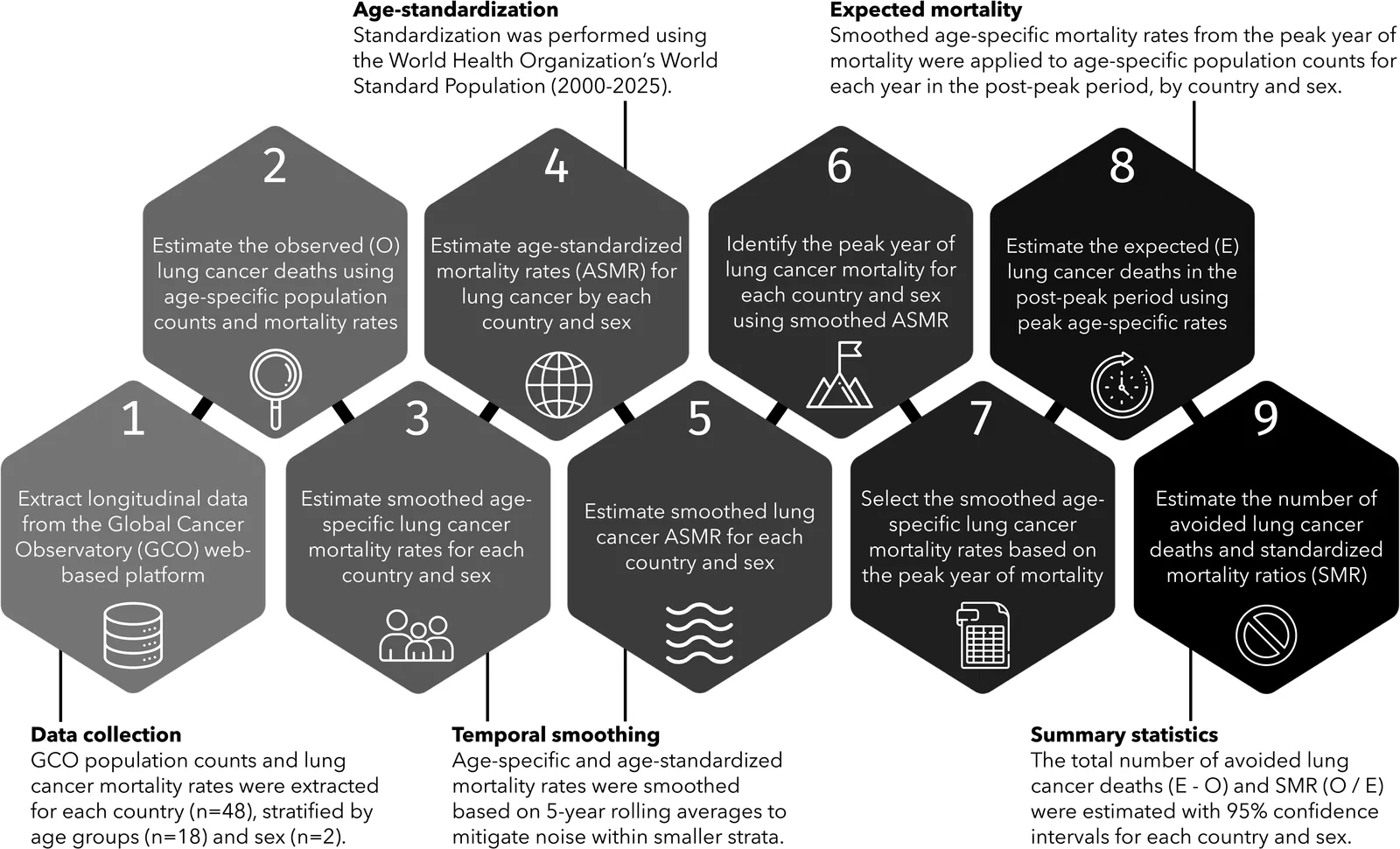
Schematic for the data collection and statistical analysis performed in this study

## Results

We analyzed lung cancer mortality data from 48 countries for both males and females from 1950 to 2019. Data were not available for all countries for all years, see Supplemental Figure 1 for data availability by country and sex. The median number of years of data available was 56 (IQR: 35 to 67 years). There were 11 countries in the Americas (North and South), 31 countries in Europe, 4 countries in Asia, and 2 countries in Oceania. Forty-one countries (85.4%) were very high Human Development Index (HDI > 0.8), six countries (12.5%) were high HDI (HDI between 0.700 and 0.799), and one country (2.1%) was medium HDI (HDI between 0.550 to 0.699). There were no countries from Africa and limited representation of Asia in this study.

### Peak Age-Standardized Lung Cancer Mortality Rates

ASMR were standardized to the WHO World Standard population to be comparable across all countries and to identify the peak year of lung cancer mortality for each country and sex, if mortality has peaked. ASMR over time by country and sex are presented in Figure 2. ASMR has peaked for all forty-eight countries for males and for all but eleven countries for females (23% of countries yet to peak). However, in countries where lung cancer mortality had peaked for both sexes, the peak ASMR was, on average, 36.3 per 100,000 higher for males, and occurred an average of 20.5 years earlier for males (1988) than for females (2008). At their peaks, the closest ASMR were for Ecuador (8.0 per 100,000 for males and 4.3 per 100,000 for females) and the furthest apart were for Czechia (87.3 per 100,000 for males and 15.7 per 100,000 for females). The highest ASMR for males across all countries was 91.3 per 100,000 in Hungary in 1996. For females, the highest ASMR across all countries was 34.8 per 100,000 in Scotland in 2008. For males, the earliest country to peak for lung cancer mortality was Finland in 1970 (73.2 per 100,000) and the latest country to peak was Romania in 2010 (52.5 per 100,000). For females, the earliest country to peak (among those that have peaked) was Costa Rica in 1985 (6.34 per 100,000) and the latest to peak was Austria in 2018 (15.9 per 100,000). The eleven countries that had not yet peaked for females were France, Germany, Greece, Italy, Lithuania, Malta, Romania, Slovenia, Spain, Northern Ireland, and Uruguay. The peak year of lung cancer mortality and corresponding ASMR are presented in Table 1.

**Figure 2.**
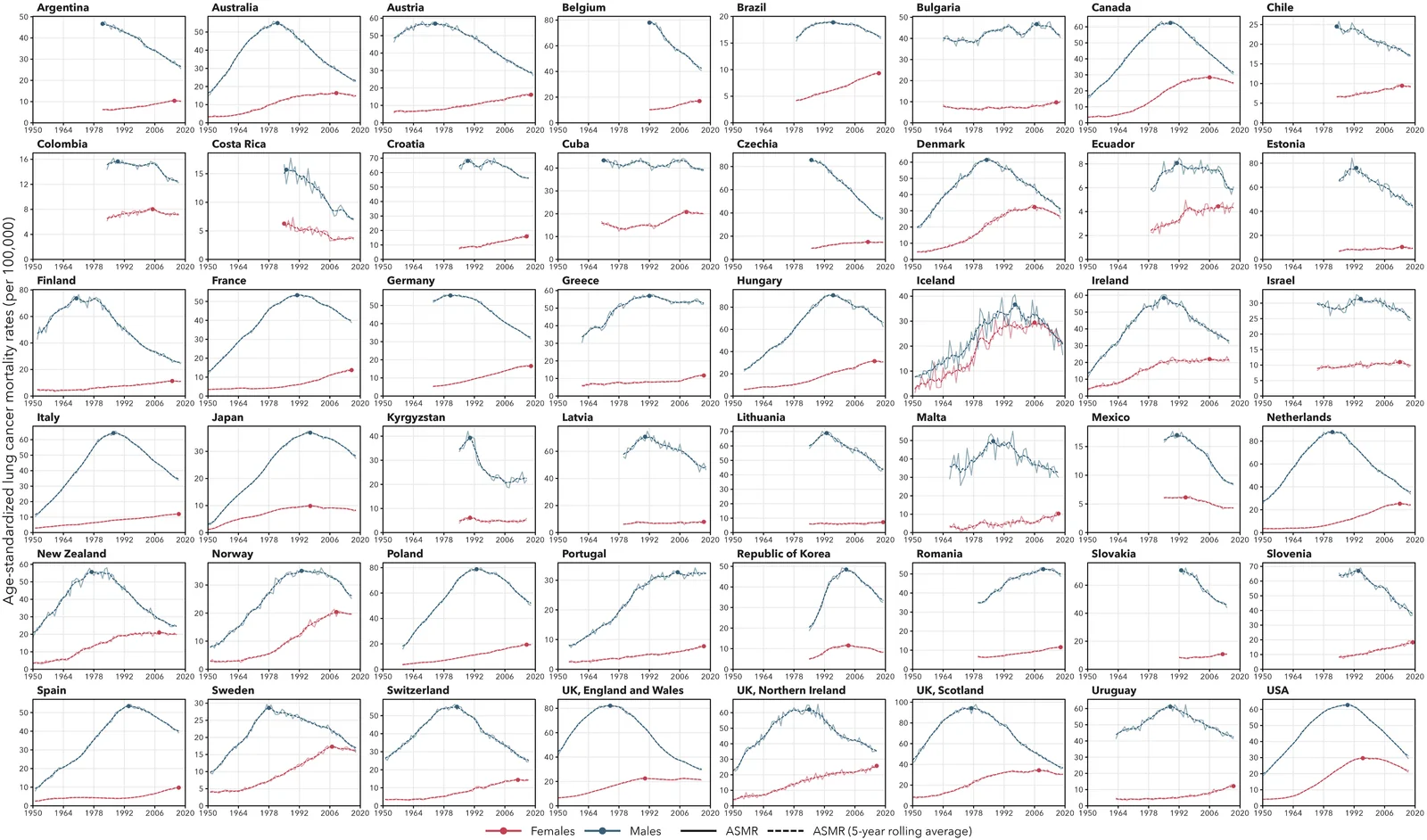
Age-standardized mortality rates (ASMR) for lung cancer from 1950 to 2019 for 48 countries. ASMR were standardized to the World Health Organization’s (WHO) World Standard Population (WHO 2000-2025). ASMR are shown separately for women (red) and men (blue). Both the ASMR (solid line) and the five-year rolling average (dashed line) are presented. The peak five-year rolling average for the ASMR is indicated with a point

**Table 1.** Number of observed, expected, and avoided lung cancer deaths and standardized mortality ratios (SMR) across 48 countries for the period after age-standardized lung cancer mortality rates peaked for each sex

​	Sex	Peak Year	Peak ASMR^a^	Cancer deaths				
				Period	Observed	Expected^b^	Avoided^c^	SMR^d^
Argentina	Female	2015	10.5	2016-2018	9,409	9,528	121	0.99 (0.97-1.01)
	Male	1982	45.9	1983-2018	231,378	295,468	64,240	0.78 (0.78-0.79)
Australia	Female	2009	16.4	2010-2018	29,902	31,109	1,262	0.96 (0.95-0.97)
	Male	1982	55.1	1983-2018	169,199	257,886	88,687	0.66 (0.65-0.66)
Austria	Female	2018	15.9	2019-2019	1,648	1,587	0	1.04 (0.99-1.09)
	Male	1974	56.6	1975-2019	109,114	147,504	38,389	0.74 (0.74-0.74)
Belgium	Female	2015	17.0	2016-2016	1,936	1,954	18	0.99 (0.95-1.04)
	​	1992	79.0	1993-2016	121,176	162,815	41,715	0.74 (0.74-0.75)
Brazil	Female	2017	9.3	2018-2018	12,353	12,245	0	1.01 (0.99-1.03)
	Male	1996	18.7	1997-2018	281,694	305,564	23,934	0.92 (0.92-0.93)
Bulgaria	Female	2016	9.1	2017-2018	1,543	1,490	2	1.04 (0.98-1.09)
	Male	2007	46.7	2008-2018	30,126	31,104	1,251	0.97 (0.96-0.98)
Canada	Female	2006	28.8	2007-2017	98,731	101,769	3,114	0.97 (0.96-0.98)
	Male	1988	63.3	1989-2017	294,875	401,675	106,935	0.73 (0.73-0.74)
Chile	Female	2014	9.4	2015-2018	5,280	5,368	103	0.98 (0.96-1.01)
	Male	1984	24.2	1985-2018	47,098	56,983	9,965	0.83 (0.82-0.83)
Colombia	Female	2005	8.1	2006-2017	20,399	22,110	1,728	0.92 (0.91-0.94)
	Male	1989	15.6	1990-2017	56,580	62,994	6,421	0.90 (0.89-0.91)
Costa Rica	Female	1985	6.3	1986-2017	2,451	3,521	1,085	0.70 (0.67-0.72)
	Male	1986	13.2	1987-2017	4,963	7,561	2,614	0.66 (0.64-0.67)
Croatia	Female	2016	15.0	2017-2017	852	771	0	1.11 (1.03-1.18)
	Male	1989	67.5	1990-2017	57,559	60,703	3,341	0.95 (0.94-0.96)
Cuba	Female	2009	20.8	2010-2017	15,533	15,826	331	0.98 (0.97-1.00)
	Male	1971	43.2	1972-2017	114,391	122,827	8,484	0.93 (0.93-0.94)
Czechia	Female	2012	15.7	2013-2019	12,312	12,371	164	1.00 (0.98-1.01)
	Male	1986	87.3	1987-2019	139,825	202,446	62,621	0.69 (0.69-0.69)
Denmark	Female	2006	33.3	2007-2018	20,830	21,814	1,046	0.95 (0.94-0.97)
	Male	1984	61.3	1985-2018	68,231	89,043	20,852	0.77 (0.76-0.77)
Ecuador	Female	2010	4.3	2011-2017	2,267	2,305	90	0.98 (0.94-1.02)
	Male	1991	8.0	1992-2017	8,734	9,765	1,064	0.89 (0.88-0.91)
Estonia	Female	2014	10.4	2015-2019	869	913	57	0.95 (0.89-1.02)
	Male	1993	73.2	1994-2019	13,827	17,564	3,737	0.79 (0.77-0.80)
Finland	Female	2014	11.6	2015-2018	3,127	3,161	41	0.99 (0.96-1.02)
	Male	1970	73.2	1971-2018	74,735	121,565	46,914	0.61 (0.61-0.62)
France	Female	​	​	​	​	​	​	​
	Male	1991	53.6	1992-2016	534,440	603,984	69,665	0.88 (0.88-0.89)
Germany	Female	​	​	​	​	​	​	​
	Male	1981	55.4	1982-2018	1,049,622	1,311,767	262,354	0.80 (0.80-0.80)
Greece	Female	​	​	​	​	​	​	​
	Male	1992	56.0	1993-2017	126,318	132,615	6,403	0.95 (0.95-0.96)
Hungary	Female	2015	32.0	2016-2019	13,612	13,502	17	1.01 (0.99-1.03)
	Male	1996	91.3	1997-2019	128,531	145,392	16,868	0.88 (0.88-0.89)
Iceland	Female	2006	30.4	2007-2019	905	982	87	0.92 (0.86-0.98)
	Male	1997	40.6	1998-2019	1,363	1,713	354	0.80 (0.75-0.84)
Ireland	Female	2006	22.1	2007-2015	6,626	6,619	110	1.00 (0.98-1.03)
	Male	1985	60.4	1986-2015	30,334	40,031	9,737	0.76 (0.75-0.77)
Israel	Female	2013	10.4	2014-2018	3,239	3,375	165	0.96 (0.93-0.99)
	Male	1995	31.2	1996-2018	24,434	26,841	2,413	0.91 (0.90-0.92)
Italy	Female	​	​	​	​	​	​	​
	Male	1987	65.2	1988-2017	760,192	934,706	174,999	0.81 (0.81-0.82)
Japan	Female	1997	9.8	1998-2018	379,551	395,260	15,863	0.96 (0.96-0.96)
	Male	1997	36.8	1998-2018	985,005	1,083,332	98,328	0.91 (0.91-0.91)
Kyrgyzstan	Female	1990	7.0	1991-2016	2,472	3,130	658	0.79 (0.76-0.82)
	Male	1990	38.3	1991-2016	8,964	15,096	6,137	0.59 (0.58-0.61)
Latvia	Female	2017	7.9	2018-2018	233	213	0	1.09 (0.96-1.24)
	Male	1990	67.6	1991-2018	24,127	28,002	3,941	0.86 (0.85-0.87)
Lithuania	Female	​	​	​	​	​	​	​
	Male	1993	67.8	1994-2019	30,132	36,313	6,200	0.83 (0.82-0.84)
Malta	Female	​	​	​	​	​	​	​
	Male	1987	51.8	1988-2017	3,348	4,177	845	0.80 (0.77-0.83)
Mexico	Female	1995	6.0	1996-2017	49,161	61,463	12,370	0.80 (0.79-0.81)
	Male	1991	16.6	1992-2017	111,144	155,957	44,917	0.71 (0.71-0.72)
Netherlands	Female	2013	25.1	2014-2018	21,518	21,547	170	1.00 (0.99-1.01)
	Male	1982	88.6	1983-2018	240,490	364,245	123,807	0.66 (0.66-0.66)
New Zealand	Female	2008	22.3	2009-2016	6,237	6,347	141	0.98 (0.96-1.01)
	Male	1977	55.4	1978-2016	34,664	50,486	15,888	0.69 (0.68-0.69)
Norway	Female	2009	18.8	2010-2016	6,726	6,595	0	1.02 (1.00-1.04)
	Male	1993	35.4	1994-2016	27,182	28,670	1,642	0.95 (0.94-0.96)
Poland	Female	2016	19.2	2017-2018	15,929	15,755	0	1.01 (1.00-1.03)
	Male	1993	79.1	1994-2018	401,965	472,426	70,646	0.85 (0.85-0.85)
Portugal	Female	2017	7.7	2018-2018	1,038	1,013	0	1.03 (0.96-1.09)
	Male	2005	32.8	2006-2018	38,296	38,400	538	1.00 (0.99-1.01)
Republic of Korea	Female	2003	11.5	2004-2019	68,360	77,521	9,178	0.88 (0.88-0.89)
	Male	2002	48.9	2003-2019	198,776	245,482	46,706	0.81 (0.81-0.81)
Romania	Female	​	​	​	​	​	​	​
	Male	2010	52.5	2011-2018	62,797	63,051	644	1.00 (0.99-1.00)
Slovakia	Female	2012	10.4	2013-2014	1,114	1,097	0	1.02 (0.96-1.08)
	Male	1993	71.4	1994-2014	36,108	45,402	9,336	0.80 (0.79-0.80)
Slovenia	Female	​	​	​	​	​	​	​
	Male	1994	67.6	1995-2019	19,273	25,370	6,118	0.76 (0.75-0.77)
Spain	Female	​	​	​	​	​	​	​
	Male	1994	54.0	1995-2017	381,166	426,433	45,519	0.89 (0.89-0.90)
Sweden	Female	2007	17.5	2008-2018	19,444	18,983	11	1.02 (1.01-1.04)
	Male	1978	28.6	1979-2018	73,396	88,814	15,473	0.83 (0.82-0.83)
Switzerland	Female	2012	14.4	2013-2017	6,182	6,076	35	1.02 (0.99-1.04)
	Male	1984	53.4	1985-2017	68,293	99,718	31,436	0.68 (0.68-0.69)
UK, England and Wales	Female	1990	22.3	1991-2016	314,112	297,924	0	1.05 (1.05-1.06)
	Male	1974	83.2	1975-2016	887,828	1,339,311	451,482	0.66 (0.66-0.66)
UK, Northern Ireland	Female	​	​	​	​	​	​	​
	Male	1985	64.8	1986-2016	16,267	21,561	5,333	0.75 (0.74-0.77)
UK, Scotland	Female	2008	34.8	2009-2019	21,972	22,770	879	0.96 (0.95-0.98)
	Male	1977	88.9	1978-2019	103,823	151,976	48,292	0.68 (0.68-0.69)
USA	Female	1996	29.7	1997-2017	1,430,242	1,518,686	99,874	0.94 (0.94-0.94)
	Male	1989	62.5	1990-2017	2,484,302	3,305,351	821,904	0.75 (0.75-0.75)
Uruguay	Female	​	​	​	​	​	​	​
	Male	1988	62.5	1989-2017	28,931	34,410	5,534	0.84 (0.83-0.85)

Abbreviations: ASMR, age-standardized mortality rate; CI, confidence interval; SMR, standardized mortality ratios; UK, United Kingdom.

^a^Age-standardized mortality rates are per 100,000 people based on direct standardization to the World (WHO 2000-2025) Standard population. We used 5-year rolling average ASMR to identify peak year of mortality but observed ASMR are reported in this table.

^b^Expected cancer deaths were estimated based on applying age-specific mortality rates from the peak year to age-specific population counts for every year in the period following the year of peak mortality.

^c^If mortality rates only peaked recently or they have been relatively stable post-peak mortality, then the number of observed lung cancer deaths may still exceed the expected lung cancer deaths. In these situations, the number of avoided lung cancer deaths is considered as zero until the observed deaths are below the expected deaths (i.e., no reductions in expected have been observed to date).

^d^Standardized mortality ratios (SMR) were estimated as the ratio between the expected and observed number of deaths. Confidence intervals (95%) were estimated using the Vandenbroucke method.

### Number of Avoided Lung Cancer Deaths

The period since lung cancer mortality peaked and the number observed lung cancer deaths, expected lung cancer deaths, and avoided lung cancer deaths are presented in Table 1. Figure 3 presents the difference between the observed and expected lung cancer deaths by country and sex. In total there were 13,353,133 lung cancer deaths, 16,401,200 expected lung cancer deaths, and 3,083,344 avoided lung cancer deaths for males and females combined across all countries. For males among the 48 countries with mortality rates that peaked, there were 10,745,016 lung cancer deaths, 13,674,498 expected lung cancer deaths, and 2,934,625 avoided lung cancer deaths. For females among the 37 countries with mortality rates that peaked, there were 2,608,116 lung cancer deaths, 2,726,702 expected lung cancer deaths, and 148,719 avoided lung cancer deaths. Ninety-five percent of all avoided lung cancer deaths were among males. There were approximately 20 times more lung cancer deaths avoided for males than females. The median number of avoided deaths were 15,681 and 103 for males and females, respectively. The countries with the three largest amounts of avoided lung cancer deaths were USA (821,904; 1990-2017), England and Wales (451,482; 1975-2016), and Germany (262,354; 1982-2018) for males, and USA (99,874; 1997-2017), Japan (15,863; 1998-2018), and Mexico (12,370; 1996-2017) for females. These three countries with the largest total number of avoided deaths account for 52.3% and 86.1% of the total avoided lung cancer deaths for males and females, respectively. See Figure 4 for the lung cancer deaths avoided by country and sex. Per capita in the most recent year of avoided deaths, males in the Netherlands (2018) and females in the USA (2017) were avoiding 1,055 and 127 lung cancer deaths per 100,000 persons.

**Figure 3.**
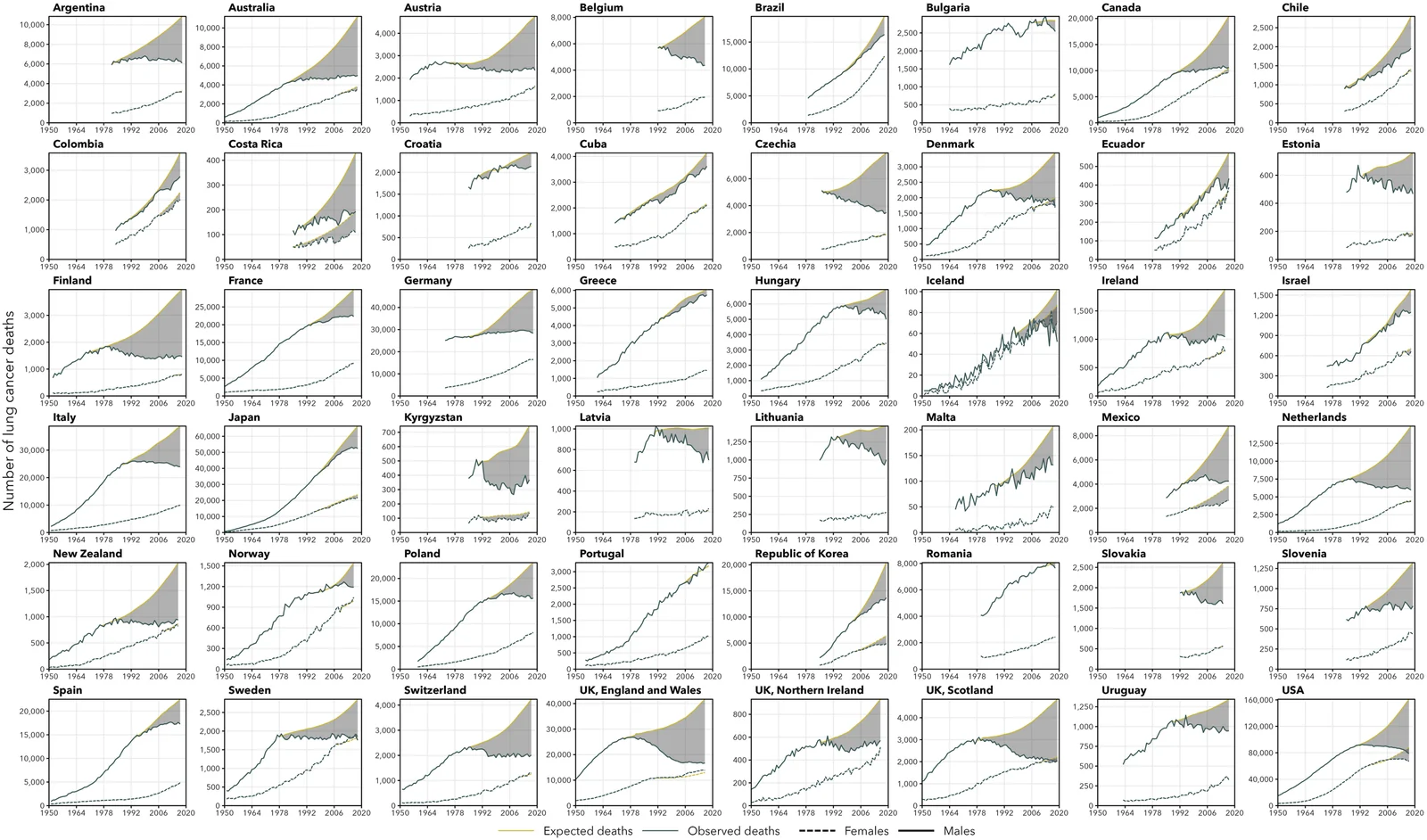
Avoided lung cancer deaths from 1950 to 2019 for 48 countries. Observed (green) and expected (yellow) lung cancer deaths are presented over time separately for women (dashed line) and men (solid line). The shaded grey region is the total amount of avoided lung cancer deaths since age-standardized lung cancer mortality rates peaked based on the five-year rolling average

**Figure 4.**
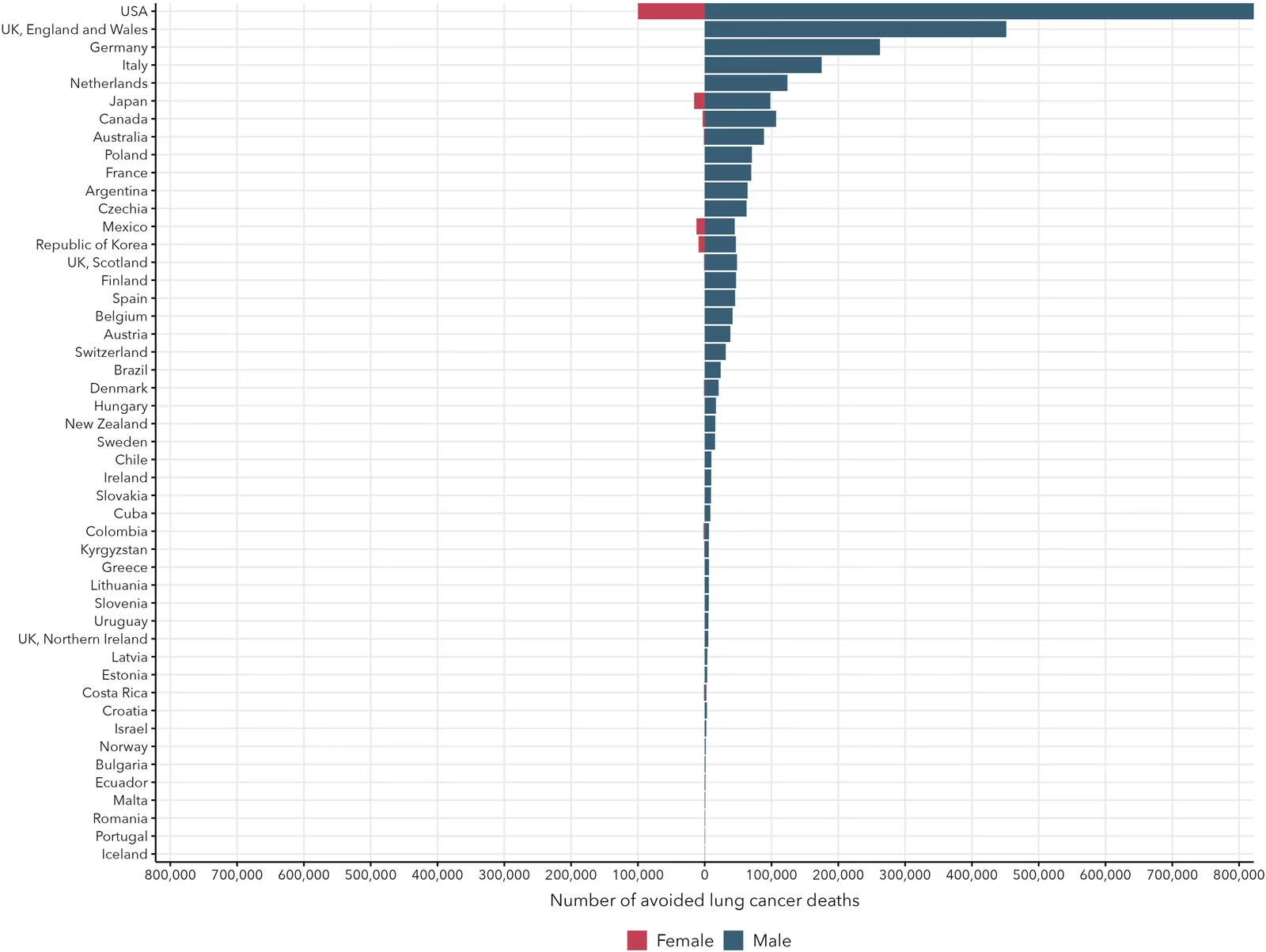
Total number of avoided lung cancer deaths by country and sex. The number of avoided deaths in each year since lung cancer mortality rates peaked are summed separately for women (red) and men (blue). Countries are ordered from top-to-bottom based on the total descending number of avoided lung cancer deaths for both sexes

### Relative Reductions in Lung Cancer Mortality

The total number of avoided lung cancer deaths is a function of the progress made in reduction lung cancer mortality and the population size of the country. In this section we will focus on the relative improvements in lung cancer mortality by country and sex. SMR for the relative reduction in lung cancer deaths over the post-peak period are presented in Figure 5 and Table 1. Forty-six (96%) and fourteen (29%) countries had significant relative reductions in lung cancer deaths for males and females, respectively. Over the entire post-peak period, fourteen countries had a relative reduction of more than 25% for males while only one country did for females (Costa Rica). In the most recent year of available data, there were fourteen countries that avoided more than 50% of expected lung cancer deaths for males, which included: Australia, Austria, Costa Rica, Czechia, Denmark, Finland, Kyrgyzstan, Mexico, Netherlands, New Zealand, Switzerland, England and Wales, Scotland, and USA (Supplemental Figure 2).

**Figure 5.**
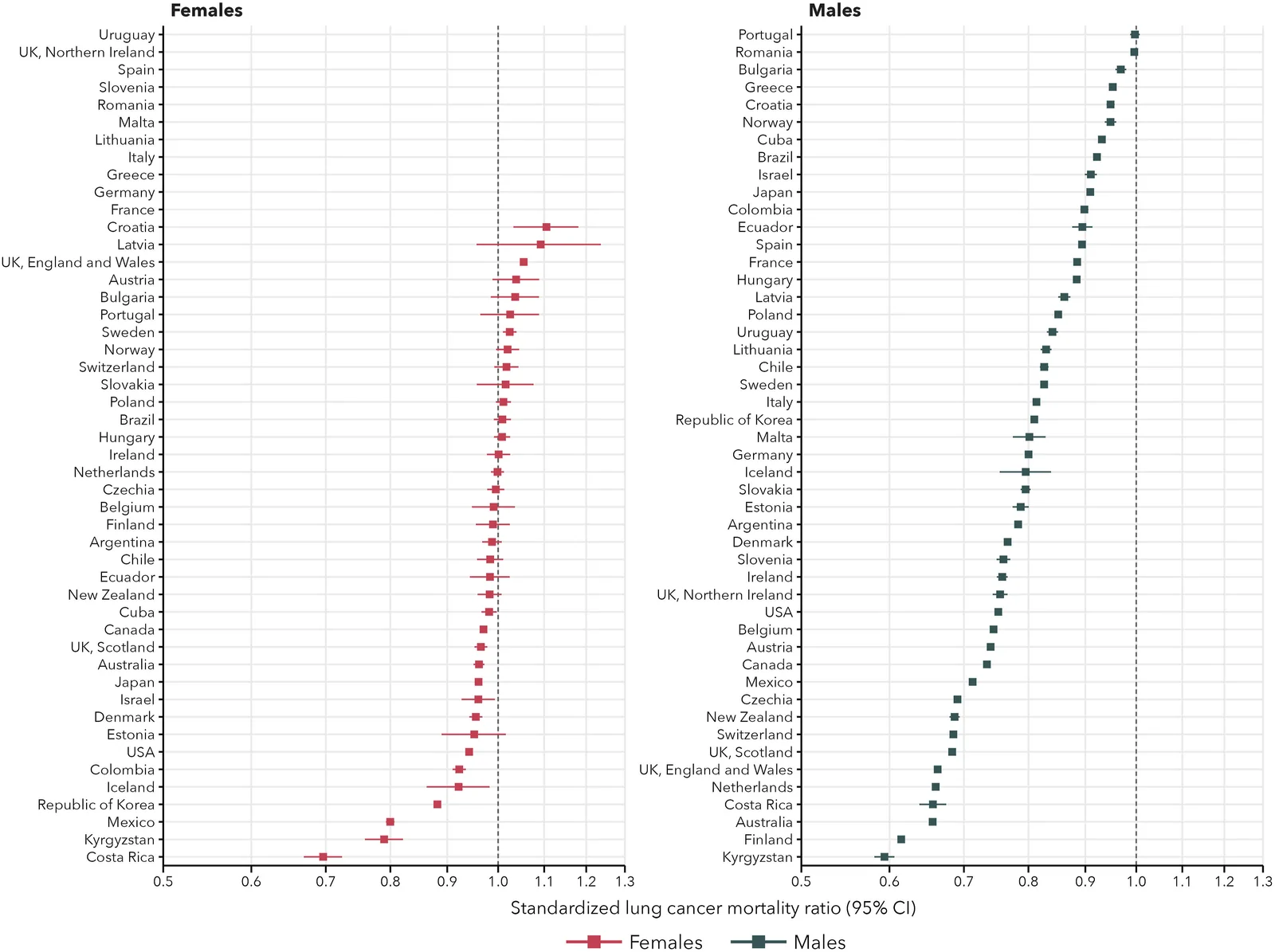
Standardized mortality ratios (SMR) for lung cancer separately by country and sex. SMR and 95% confidence intervals (CI) are presented for females (red) and males (blue). Countries are ordered top-to-bottom based on the ascending relative reduction in lung cancer mortality. Age-standardized lung cancer mortality rates have not yet peaked for countries without plotted estimates

When comparing the relative reductions in lung cancer deaths (avoided deaths divided by expected deaths) between males and females for the most recent year of data (i.e., the ratio of relative reductions for males to females), the countries closest to sex parity for avoided lung cancer deaths were Costa Rica (1.22:1), Republic of Korea (1.37:1), Mexico (1.65:1), Columbia (2.00:1), and USA (2.15:1). There are eleven countries where lung cancer mortality has not yet peaked for females, fourteen countries that have only observed reductions in males but not females despite recent peaks in mortality (Austria, Brazil, Bulgaria, Croatia, Czechia, Ecuador, England and Wales, Hungary, Latvia, Norway, Polan, Slovakia, Sweden, Switzerland), one country that has not observed reductions yet for either sex despite peaks (Portugal), and twenty-two countries where relative reductions in expected mortality have been observed for both sexes.

For the eleven and fourteen countries where mortality has not peaked for females or no avoided deaths have accrued yet, the ratio of males to females is infinite. Among the twenty-two countries where relative reductions have been observed for both sexes, there are six countries with ratios of 10-fold or larger for the relative reductions in lung cancer deaths for males to females, including Finland (92.4:1), Netherlands (59.1:1), Belgium (49.9:1), Ireland (16.4:1), Argentina (12.8:1), and Kyrgyzstan (11.2:1). The largest absolute differences in percent reductions between males and females were for Finland (62.4%; 63.1% versus <1%), England and Wales (60.0%; 60.0% versus 0%), and Netherlands (59.0%; 59.0% versus <1%), and the smallest differences were for Republic of Korea (9.0%; 32.9% versus 23.9%), Cuba (9.8%; 12.5% versus 2.7%), and Costa Rica (10.0%; 55.0% versus 45.1%).

## Discussion

In this study, we found that a considerable number of lung cancer deaths have been avoided over the last several decades, however, this progress has not been observed across all countries assessed in this study or equally for both males and females. For females, only three countries have avoided more than 10,000 lung cancer deaths in this study and these three countries account for nearly 90% of all avoided deaths across the 48 countries. For males, many countries have avoided a considerable number of lung cancer deaths and 90% of all avoided death are attributed to 17 countries. In the paragraphs that follow, we will describe some of the primary reasons that have likely driven the progress in reducing lung cancer mortality that has been observed in some countries.

The primary driver for the reduction in lung cancer mortality over the last several decades is due to reduction to tobacco use and cigarette smoking. The World Health Organization (WHO) have developed their Framework Convention on Tobacco Control to provide guidelines for countries to implement and manage tobacco control.^12^ To help realize this goal, they introduced the MPOWER measures which can help countries implement effective interventions to reduce the tobacco demand.^13^ These measures help to understand the key factors which affect smoking prevalence around the world. The MPOWER measures are: M, Monitoring tobacco use; P, Protecting people from tobacco smoke; O, Quitting tobacco (i.e., cessation programs); W, Warning about the dangers of tobacco; E, Enforcing tobacco advertising, promotion & sponsorship bans; R, Raising taxes on tobacco. These provide the framework for achieving some of the most effective ways to reduce tobacco consumption.

There are several factors that have contributed to the decline in smoking observed in many countries. Monitoring tobacco use is critical to understanding the temporal trends globally. According to The Tobacco Atlas, there are still more than one billion smokers in the world. Despite smoking prevalence declining from 22.7% in 2007 to 17% in 2021, smoking remains a serious public health issue and a cause of many morbidities including lung cancer.^14^ Long term smoking data over the period covered by this study were not readily available for most countries included in this study. Most lung cancers are caused by smoking and the reductions in smoking seen in many of these countries are likely the primary factor contributing to avoided lung cancer deaths. In the US, for example, 42.4% of adults smoked in 1965 which dropped by 73% to 11.6% by 2022.^15^ For Canada, 49.5% of Canadians smoked in 1965 and this declined to 10% in 2020.^16^ Similar trends have been observed for countries where similar peaks in lung cancer mortality were observed. Tobacco taxation (i.e., sin tax) is an important deterrent for individuals to purchase cigarettes due to the increased financial burden of purchasing tobacco products.^17,18^ These taxes often are reinvested by the government into other public health initiatives such as smoking cessation programs, mitigating healthcare costs, and anti-smoking campaigns. In addition, over many decades the availability of healthy information and counter-advertising have aimed at helping people make informed decisions about the potential consequences for smoking. In some countries, restrictions and bans on smoking in public places have also been implemented to limit passive smoke exposure.^19,20^

Generational bans on the purchase of tobacco products are a more modern approach to reducing tobacco consumption.^21,22^ These bans prohibit tobacco sales to any individuals born after a certain year. In 2021, the Massachusetts city of Brookline in the USA implemented this policy for anyone born after January 1^st^, 2000.^23^ This was the first US city to implement this type of ban at the local level. In 2022, New Zealand was the first country to implement a national-level generational smoking ban for those born on or after January 1^st^, 2009, passed under the Smokefree Environments and Regulated Products Amendment Act.^24^ However, the subsequent government repealed this in 2024.^24^ Similar policies were proposed or are under consideration in the UK and Malaysia. The UK introduced the Tobacco and Vapes Bill in April 2024 which would raise the purchasing age for tobacco products by one year each year starting with those born in or after 2009.^25^ Malaysia has proposed a Generational Endgame ban for those born after 2007 in the Control of Smoking Products for Public Health Bill, however, this ban faced considerable hurdles and was dropped in 2023.^26^ Many more countries are considering ways to create smoke-free generations, but considerable hurdles remain for widescale support and implementation.

The ASMR for lung cancer had not yet peaked or peaked only recently for females across many countries in this study. Even among countries where female ASMR had peaked, they typically did not reach the peak rates observed among males and often peaked several decades after male ASMR had peaked. One of the major reasons for these observed differences in peak lung cancer ASMR is due to differences in the social diffusion of tobacco use which affected the timing of when males and females entered the smoking epidemic. Males consistently had higher smoking prevalence than females globally throughout most of the 20th century, often by considerable margins.^27-29^ This gap was largest during the early and mid-20th century, particularly in countries where smoking was widespread among males before becoming socially acceptable for females.^28^ The gap was generally narrower in many high-income countries and remained larger in low- and middle-income countries.^28^ In many countries, peak smoking prevalence for females occurred decades after the peak for males,^27,30,31^ which is reflected in lung cancer ASMR for females also peaking decades later than for males or not yet peaking in some countries. These historical patterns reflect differences in the timing of smoking uptake and cessation between sexes which were influenced by social norms, marketing, economic development, and tobacco-control policies.^30^

Much of the discussion has focused on the reduction in lung cancer mortality rates that are likely driven by primary prevention of lung cancer attributable to reductions in cigarette smoking and tobacco consumption. However, advances in secondary and tertiary lung cancer prevention have undoubtedly contributed to the progress and avoided deaths described herein. Several large, randomized screening trials have demonstrated that regular screening with low-dose computed tomography can reduce lung cancer mortality significantly. The National Lung Screening Trial (NLST) found a 20% reduction in lung cancer mortality at six years of follow-up and this led to the subsequent development of the United States Preventive Services Task Force (USPSTF) recommendation statement for lung cancer screening in 2013.^32^ Following the recommendation from the USSPTF (in 2013) and the Centers for Medicare & Medicaid (in 2015), lung cancer screening was available in the US and likely has contributed to some of the reduction in lung cancer mortality in the US but to a considerably lesser degree than reductions in smoking.

The rollout of lung cancer screening elsewhere in the world was more slowly adopted until the readout of the Dutch-Belgian (NELSON) trial.^33^ The NELSON trial shared their results in 2020, where they reported a 24% and 33% reduction in lung cancer mortality at 10-years for males and females, respectively. Since then, more regions and countries have initiated pilot or organized lung cancer screening programs, however, the benefits of these initiatives would be modest at the population level or not yet even been realized in the period of this study. In addition to early cancer detection initiatives, advances in the treatment and clinical management of lung cancers have contributed to increases in survival and reductions in lung cancer mortality.^34-37^ Modern therapies that target lung cancer molecular subtypes (e.g., EGFR TKIs) have led to higher survival. However, much of these advances and improvements are more recent and would not account for the progress since the 70’s and 80’s for some countries or the sex differences observed in this study.

Several factors beyond tobacco control policies may have influenced the lung cancer mortality trends observed in this study. Differences in historical smoking prevalence and intensity, environmental exposures, air quality regulations, occupational safety measures, and other country-specific factors may all contribute to variations in mortality patterns. In addition, because lung cancer develops over decades, changes in mortality rates may lag substantially behind the implementation of public health policies. Consequently, the temporal trends described here should not be interpreted as direct evidence of the effectiveness of specific policies but rather as the result of multiple interacting factors that have evolved over time.

This study has several strengths. First, this study used nearly 70 years of detailed cancer mortality data from 48 countries around the world. This is one of the largest studies to try to quantify the progress made in reducing lung cancer mortality and how many fewer lung cancer deaths may have occurred due to this progress. However, there are several potential limitations to this study that should be considered. First, the estimation of expected deaths implicitly assumes that the at-risk pool remains proportional over time. Second, the countries that have contributed the data necessary for this study are predominantly from countries in Europe and the Americas. There are no African countries in this study and only limited representation of Asian countries. In addition, most of the countries included in this study are considered high or very high HDI, which is not representative of all the countries in the world. Smoking rates, air pollution, and many other lung cancer risk factors are strongly correlated to HDI and future studies with countries from a wider set of developed and developing countries are warranted. An additional limitation is that the countries included in this analysis may be at different stages of the tobacco epidemic. Given the substantial lag between smoking exposure and lung cancer mortality, observed mortality trends reflect historical smoking patterns and the cumulative effects of tobacco control measures rather than the current effectiveness of tobacco control policies alone. Consequently, cross-country differences in mortality trends should be interpreted cautiously, as they may arise from differences in smoking histories, tobacco epidemic stage, and other population-specific factors. Moreover, longitudinal comparisons of cancer mortality across countries over extended periods should be interpreted with some caution, as temporal and geographic differences in diagnostic coding practices, accuracy and completeness of mortality reporting, and the coverage of cancer registries may affect observed trends. In this study, avoided deaths only started to accrue once mortality rates reached their peak. However, lower smoking rates and progress in lung cancer mortality and avoided deaths likely began prior to peak mortality so our estimates may be somewhat conservative. However, by choosing mortality rates from the peak year to apply to future years, we have estimated a worst-case counterfactual scenario to estimate the potential number of deaths that could’ve occurred had substantial progress not been made. Nonetheless, this is one of the largest and most comprehensive studies, to date, to estimate the impact of cancer control on avoided lung cancer deaths across 48 countries based on over 70 years of detailed historical mortality data.

Our study provides important insights into the progress made in reducing lung cancer mortality in 48 countries over the last several decades. The substantial number of avoided lung cancer deaths highlights the importance of continued investment in cancer prevention, screening, and treatment efforts to further reduce the impact of the global leader in cancer mortality. Despite, in many countries, an increasing total number of lung cancer deaths due to aging and growing populations, lung cancer mortality rates peaked many years ago for some countries and this has led to an avoidance of lung cancer deaths. This study highlights the great strides that have been made in lung cancer control and presents the substantial number of lives saved that can be attributed to the considerable advances made over the last several decades in prevention, screening, and treatment.

## Supplemental Material

Supplemental Material - Temporal Trends in Lung Cancer Mortality Across 48 Countries from 1950 to 2019: An Ecological Study Supplemental Material for Temporal Trends in Lung Cancer Mortality Across 48 Countries from 1950 to 2019: An Ecological Study by Matthew T. Warkentin, Yibing Ruan, Alain Tremblay and Darren R. Brenner in Cancer Control.

## Data Availability

The data analyzed for the current study are available from the Global Cancer Observatory (https://gco.iarc.fr/overtime/en).
