# Supplementary material for: Temporal Trends in Lung Cancer Mortality Across 48 Countries from 1950 to 2019: An Ecological Study
Source: Cancer Control. 2026 Oct 7;33:10732748261493115. doi: 10.1177/10732748261493115 (PMC13770036; doi:10.1177/10732748261493115)
Supplement: Supplementary file 1 [file sj-pdf-1-ccx-10.1177_10732748261493115.pdf]

SUPPLEMENTAL MATERIALS FOR:

Temporal Trends in Lung Cancer Mortality Across 48 Countries from 1950 to 2019: An Ecological Study

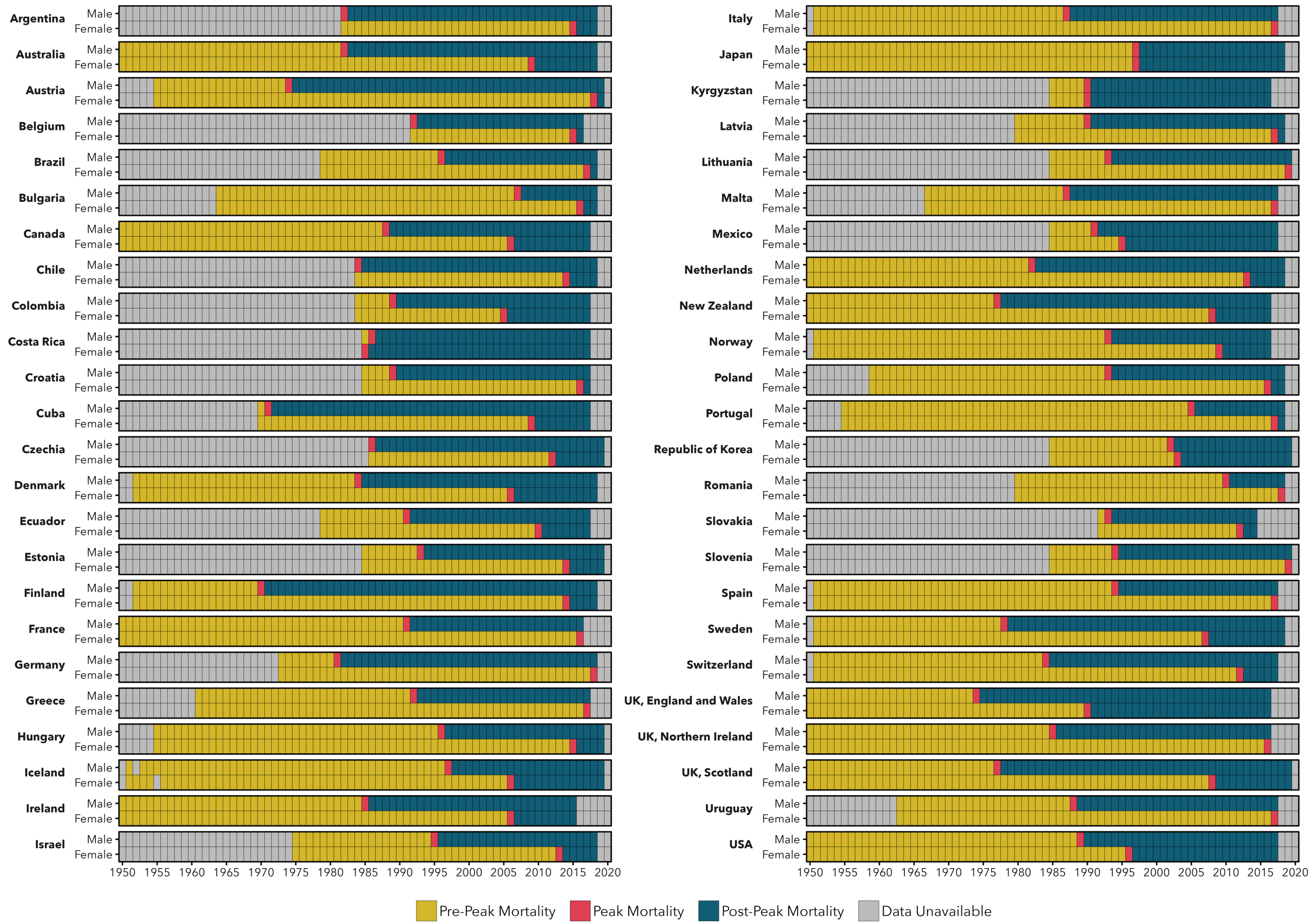

Supplemental Figure 1. Data availability before (pre-peak), at (peak), and after (post-peak) the peak year in age-standardized lung cancer mortality rates based on country and sex.

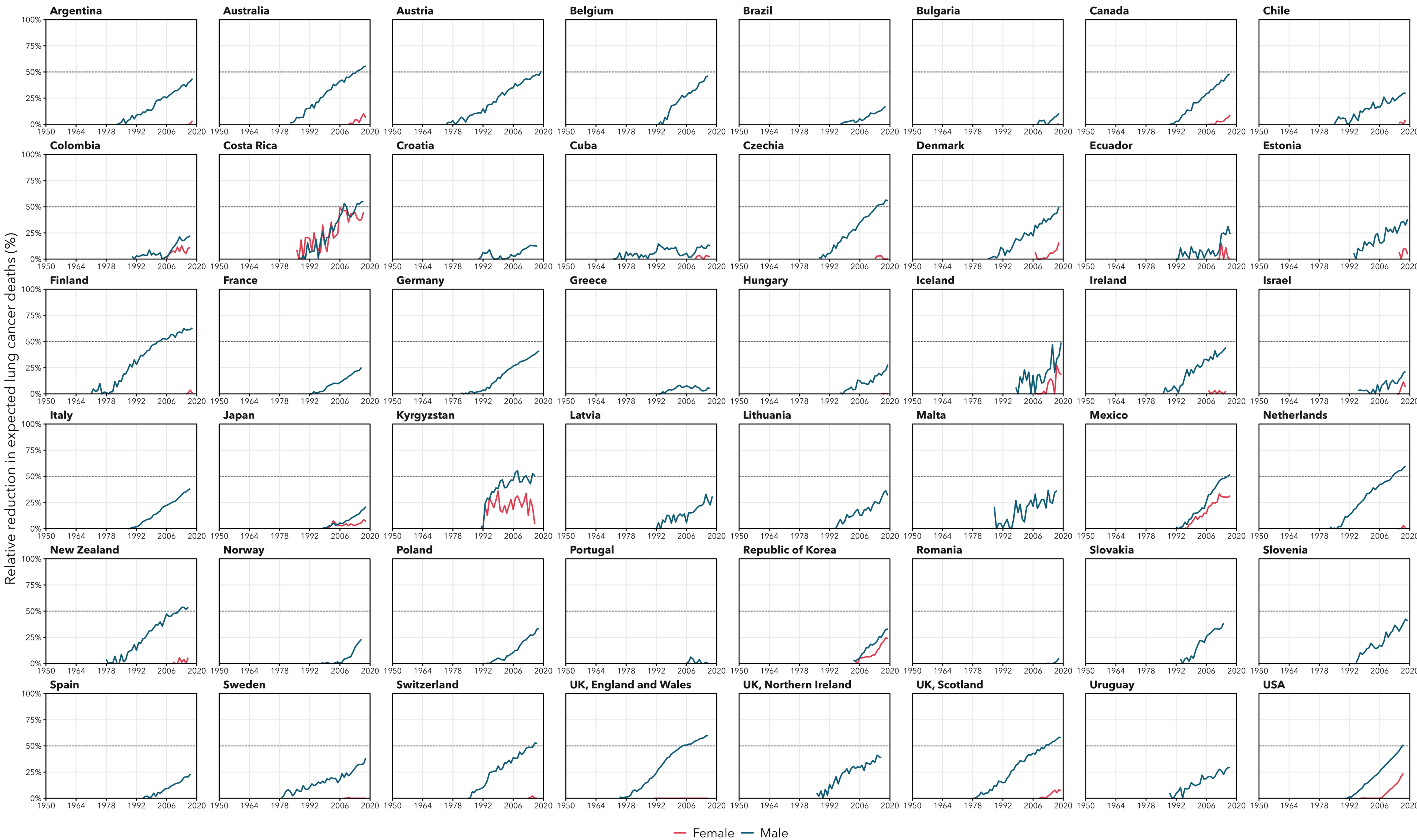

Supplemental Figure 2. Relative reduction in lung cancer deaths for each country and sex. Relative reduction is based on the ratio of avoided deaths to expected deaths.
